# Quantifying social performance: A review with implications for further work

**DOI:** 10.3389/fpsyg.2023.1124385

**Published:** 2023-04-27

**Authors:** Marcus G. Wild, Rebecca A. Cutler, Jo-Anne Bachorowski

**Affiliations:** ^1^VISN 17 Center of Excellence for Research on Returning War Veterans, Waco, TX, United States; ^2^Department of Psychology and Neuroscience, University of Texas, Austin, TX, United States; ^3^Department of Psychology, Vanderbilt University, Nashville, TN, United States

**Keywords:** social skill acquisition, social expertise, emotional intelligence, social intelligence, social performance

## Abstract

Human social performance has been a focus of theory and investigation for more than a century. Attempts to quantify social performance have focused on self-report and non-social performance measures grounded in intelligence-based theories. An expertise framework, when applied to individual differences in social interaction performance, offers novel insights and methods of quantification that could address limitations of prior approaches. The purposes of this review are 3-fold. First, to define the central concepts related to individual differences in social performance, with a particular focus on the intelligence-based framework that has dominated the field. Second, to make an argument for a revised conceptualization of individual differences in social–emotional performance as a social expertise. In support of this second aim, the putative components of a social–emotional expertise and the potential means for their assessment will be outlined. To end, the implications of an expertise-based conceptual framework for the application of computational modeling approaches in this area will be discussed. Taken together, expertise theory and computational modeling methods have the potential to advance quantitative assessment of social interaction performance.

## Introduction

There is an abundance of measures used to assess constructs that describe individual differences in social interactions. These constructs are not based on a common conceptual framework, but are instead based on a collection of frameworks that includes intelligences, traits, abilities, and social skills (e.g., Davis, 1983; Salovey and Mayer, 1990; Mayer et al., 2008; Riggio, 2010). Despite conceptual differences among these constructs, there is nonetheless significant covariance among most measures of individual differences in social interactions (Schlegel et al., 2017; Hussey and Hughes, 2020; Olderbak and Wilhelm, 2020). As a result, there is now a large, highly interconnected set of constructs with overlapping measures, all intended to assess these differences. Despite the theoretical connections and overlap among measures, the constructs themselves lack a unifying conceptual underpinning. The resulting complexity may in part mirror the natural complexity inherent to human social interactions. We posit “expertise” as an organizing concept that can account for the dynamic interplay among ability, skill, and performance. Investigators studying individual differences in skilled performance, including chess and object recognition, have arrived at the concept of *expertise* to account for consistently superior skilled performance (Ericsson, 2006). Expertise has been operationalized to model the manner in which individual differences in ability, skill, traits, intelligence, and experience interact to influence performance (e.g., Ericsson, 2004; Ackerman, 2007; Ullen et al., 2016; Logan, 2018). Following from models of expertise in other domains (Ullen et al., 2016), Figure 1 displays a simplified framework for how expertise could be used in conceptualizations of social performance.

**Figure 1:**
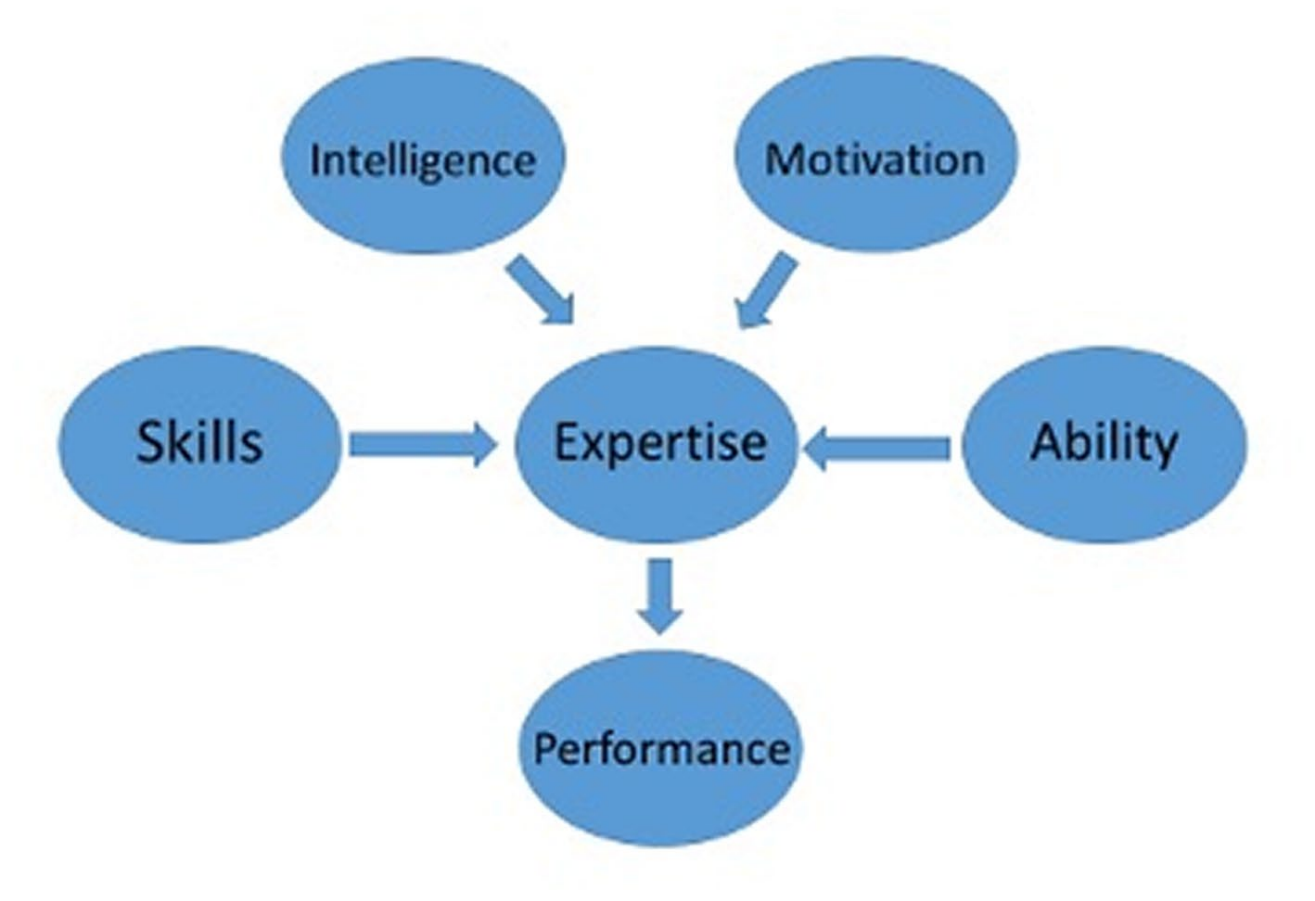
Conceptual model of the contributing factors of expertise and social performance, and their organization. Adapted from Ullen et al. (2016).

Expertise offers a framework in which abilities, skills, traits, intelligence, and experience can be combined to account for domain-specific performance. Social–emotional expertise (SEE) is the synthesis of experience, skills, and abilities that lead to individual differences in social interaction performance. The two factors that contribute to SEE are individual differences in cognition, and the quality and timing of behaviors in social interactions that affect social interaction performance (McBrien et al., 2018; Wild and Bachorowski, 2020).

The goals of this paper are to (1) review conceptual differences among constructs designed to assess individual differences in social interactions, (2) propose expertise as a unifying conceptual framework for these constructs, and (3) discuss how computational modeling and multi-dimensional representational space can be used to capture the complexities of social interactions. Toward these aims, the first section will begin with a brief review of conceptual barriers for social performance constructs followed by a discussion of the prevailing conceptual framework for individual differences in social performance: intelligence.

## Constructs used to assess individual differences in social interactions

“Individual differences” is the term given to the between-individual variance that can be observed in measures of constructs (Olderbak and Wilhelm, 2017; Richler et al., 2019). Individual differences are typically expressed along dimensions of a purported characteristic, such as extraversion or social anxiety. Notable variability in measured social interactions prompted an array of perspectives attempting to explain observed differences. The resultant “constructs” are attributes of people, and often used to conceptualize the mechanisms underlying individual differences. Construct validation is the determination of the extent to which the measures devised to test the construct are actually measuring the attribute of interest, often through the development of a “nomological network” of relations among related constructs (Cronbach and Meehl, 1955). Construct conceptualizations shape measurement methods. Individual differences in social interactions have been variously conceptualized as being attributable to differences among: (a) a set of traits, or time-stable, typical performances attributes such as emotional empathy and extraversion; (b) a set of related abilities, or maximal performance attributes that can change over time such as cognitive empathy and interpersonal accuracy; (c) a general social/emotional intelligence (ability); and (d) a collection of related social skills.

The distinction between traits and abilities is most commonly reflected in measurement methods: self-report measures are most commonly used to assess traits, and performance-based measures are optimal for abilities. However, mismatches between constructs and measurement approaches are often observed in the social interaction domain. Constructs conceptualized as traits have been measured with scales that are not reliable over time or context, constructs proposed as abilities have been measured with self-report questionnaires, and checklists of skills have been used without a construct that describes the attribute(s) being measured (e.g., Schutte et al., 1998, 2009; Wong and Law, 2002; Bar-On, 2004; Wong et al., 2004; Mikolajczak et al., 2007; Petrides, 2009). Although few attributes are solely a trait, ability, or skill, assessment of the trait components of a construct (e.g., emotional empathy) should be assessed differently from ability components of a construct (e.g., cognitive empathy). The result of mismatches between conceptualization and measurement approaches are a limitation of social performance construct validation.

The criterion problem, or the difficulty in identifying definitive criteria for measures of a construct, is another particular problem for construct validation in the social domain (Cronbach and Meehl, 1955; Thurstone, 1955). There are at least two attributes of social interactions that contribute to the criterion problem. First, social interactions are dynamic. They leverage so many aspects of behavior and cognition (e.g., nonverbal and verbal behavior, decision-making, emotion recognition, etc.) that *post hoc* justifications are easily construed—even when there is no obvious *a priori* conceptual need for the constructs to be associated. Second, there are few means by which to determine a criterion for social interactions. While measures of intelligence can have correct/incorrect responses, social interactions cannot typically be reduced to a dichotomous outcome. These two issues—liberal measurement correlation and lack of clear outcome criteria—have blurred the delineation of conceptualization and measurement, and have therefore made construct validation difficult (Olderbak and Wilhelm, 2020).

The prevailing approach to individual differences in social interactions has nonetheless been that they are the result of an intelligence-like ability. Definitions of intelligence and applications of intelligence to individual differences in social interactions are therefore examined next.

### Intelligence

The concept of intelligence has been central to thinking about individual differences in social interactions. This is the case not just in the published literature (e.g., Mayer et al., 2008, 2016; Evans et al., 2020), but in the popular press as well (e.g., Goleman, 1995, 2007). Intelligence is broadly considered to be a global cognitive ability, the extent to which a person can acquire, hold, and use information (Carroll, 1993; Neisser et al., 1996; Jensen, 1998; Schneider and McGrew, 2018). Measurement of intelligence has traditionally focused on maximum-performance ability measures like the Wechsler series of intelligence tests (e.g., Wechsler Adult Intelligence Scale-Fourth Edition; Drozdick et al., 2018). These tests assess performance on increasingly difficult tasks to measure working memory, spatial/perceptual reasoning, verbal comprehension, and processing speed (Ackerman and Heggestad, 1997). Intelligence tests have been modeled largely using hierarchical latent factor structures to reflect the theoretical assumption that intelligence is an underlying ability that affects a broad range of performance (Wechsler, 1958). However, questions remain regarding the suitability of hierarchical factor models for assessing intelligence due to concerns with reliability of models across samples, and threats to validity from the undue influence of cultural knowledge and systemic racism on test performance (see Helms, 2006; Watkins, 2017; Issarraras and Matson, 2018). These concerns extend to assessments of social cognition (Dodell-Feder et al., 2020), and to psychological research more broadly (e.g., Roberts et al., 2020). Our contemporary, albeit incomplete, understanding of intelligence nonetheless continues to posit that a single, general factor is responsible for the common variance among the domains assessed.

### Social intelligence

When seeking to explain individual differences in social interactions, intelligence has been a natural conceptual framework with which to begin, given its prevalent application to other forms of complex problem-solving (e.g., Carroll, 1993). The first attempt to apply an intelligence framework to social interactions resulted in the construct of “social intelligence” (SI; Thorndike, 1920). SI was initially defined as the ability to “understand and behave wisely” in interactions with others and was further postulated to be one of three primary aspects of intelligence (Thorndike, 1920). Threats to construct validity led research efforts to be largely abandoned less than two decades later (Thorndike and Stein, 1937). A resurgence of interest beginning in the 1980s, with focused attention given to construct validity, led to the contemporary emphasis on social problem solving and other factors that might differentiate SI from general intelligence (Sternberg and Smith, 1985; Cantor and Kihlstrom, 1986; Kihlstrom and Cantor, 2000). Further specificity in both theory and measurement paved the way for the umbrella construct of “emotional intelligence” (EI; Mayer and Salovey, 1993; Mayer et al., 2001).

Interest in SI did not, however, abate following the advent of EI. Measurement of SI has centered on self-report, with the Tromso Social Intelligence Scale, the most commonly used measure (Silvera et al., 2001; Grieve and Mahar, 2013). Although ability measures were developed early on in SI research (e.g., the George Washington University Social Intelligence Test; Moss et al., 1925), these were found to be psychometrically inadequate. Modern performance measures are essentially unavailable. Instead, items from SI self-report measures assess one’s impressions of how individuals use social information to problem-solve in social contexts (Kihlstrom and Cantor, 2000; Grieve and Mahar, 2013). This approach to assessing SI necessarily includes one’s self-efficacy regarding social problem-solving abilities. Additionally, as SI measures now typically include self-evaluations of behaviors in social situations rather than problem-solving with social information, the data captured by the most commonly used measures actually extends beyond the theoretical scope of SI and into the realm of social skills. As a result, SI, social skills, *and* social interaction self-efficacy are likely confounded in self-report measures of SI.

### Emotional intelligence

In part stemming from difficulties establishing the validity of SI, EI emerged as a more focused approach. EI was originally defined as the abilities to “accurately understand the emotions of one’s self and others,” and to use emotional information to influence one’s own thinking and the thoughts of others (Salovey and Mayer, 1990). The EI construct has since been refined, and now emphasizes the ability to reason with emotions and to use emotion to enhance thought through four problem-solving domains (Mayer et al., 2016). EI is said to meet the traditional standards for an intelligence and as a “hot” intelligence that supports reasoning abilities significant to the individual (Mayer et al., 1999, 2016). Ability measures of EI, such as the Mayer-Salovey-Caruso Emotional Intelligence Test (MSCEIT), were developed based on traditional approaches to intelligence assessment and therefore include maximum performance and accuracy scoring (Mayer et al., 1999, 2001).

Emotional intelligence assessments vary in scope and form, with self-report and non-social performance tests the most prevalent (Schutte et al., 1998; Wong and Law, 2002; Bar-On, 2004; Wong et al., 2004; Mikolajczak et al., 2007; Petrides, 2009; Schutte et al., 2009). Self-report measures have been noted to be suboptimal, and often include measures of behaviors that are either not definitionally consistent with EI, or that are designed to assess EI only in specific contexts (i.e., the workplace; Palmer et al., 2009; Gignac, 2010). The MSCEIT is the most widely used performance measure (O'Connor et al., 2019) and has therefore formed the basis of much of the construct validity of EI. The MSCEIT has been well validated, though with substantial critiques focused on subtests (e.g., the “Using Emotions” subtest) and scoring procedures (Murphy, 2006; Roberts et al., 2006; Fiori et al., 2014). The critiques of the Using Emotions subtest (i.e., the application of emotional understanding to behavior) address a central issue for EI. If EI is an intelligence, then EI assessments, as measures of intelligence, should not be expected to solely account for the application of understanding and perception to effective behavior (i.e., performance). As a result, the weakest subtest of the MSCEIT is assessing a domain outside the sole scope of EI.

While general mental abilities predict a significant amount of the variance in performance (and EI is significantly related to other measures of social performance, such as empathy), it is unreasonable to expect that these mental abilities would be perfect predictors; to form better predictions, *experience* must also be accounted for (Schmidt and Hunter, 1993; Duckworth and Seligman, 2005). Though not yet tested in adult interactions directly, evidence from social learning research indicates that we typically become better at interacting with another person the more experience we have with that person (Bandura and Walters, 1977; Heyes, 2016; Whiten, 2017; Saragosa-Harris et al., 2022; see also Smoski and Bachorowski, 2003; Bachorowski and Owren, 2008). Intelligence does not account for experience. Yet, experience is crucial for social performance.

Emotional intelligence theory originally expected that EI will increase with age (i.e., experience), a positive correlation, which has been confirmed (Mayer et al., 1999, 2001; Palmer et al., 2005). Increases over the lifespan are not true of standard intelligence, with Spearman’s law of diminishing returns for age finding that over time, the variance in cognitive ability tests is actually less saturated by *g* (Spearman, 1927). That EI does demonstrate some improvements with time could be evidence for skill acquisition, and is consistent with an expertise model. Further evidence in support of experience affecting performance on measures of EI is the result that the Flynn Effect, or the generational improvement in performance on cognitive tasks (Flynn, 1987), is not found with measures of EI (Pietschnig and Gittler, 2017). Therefore, between-individual performance does not improve over time, while within-individual performance does. This pattern is a hallmark of skill acquisition.

In an attempt to incorporate experience and temporal changes in measured EI into the EI construct, distinctions between “state” and “trait” EI have been proposed (e.g., Petrides, 2009; Zampetakis and Mitropoulou, 2022). The application of trait EI measures has introduced a conceptual muddling of the construct of EI as it was originally proposed, with empirically defined intelligence famously considered a consistent ability. While the limitations of the ability approaches to EI are noted, the conflation of ability and trait approaches is a potential driver of the significant lack of specificity in language and definition of social–emotional terminology (Olderbak and Wilhelm, 2020).

### Social skills

Individual differences in social interaction performance are not easily attributable to a single ability factor like SI or EI. Alternatively, then perhaps it is the utilization of one’s SI and EI to acquire socially-relevant skills that promotes these individual differences. In other words, one’s ability to acquire skills and experience with a particular skill arguably shapes the quality of social interactions. In this regard, skills are behaviors that are demand-specific, have a degree of correct and incorrect outcomes, and occur in visuospatial, motor, and social domains (McDonald, 1965; Welford, 1980; Downing, 1982). Non-clinical assessments of social skills include the Riggio Social Skills Inventory (Riggio, 1986), the Teenage Inventory of Social Skills (Inderbitzen and Foster, 1992), and the Interpersonal Competence Questionnaire (Buhrmester et al., 1988). These scales are well-validated, widely used, and capture specific verbal and non-verbal behaviors. Examples of social skills assessed by these measures include comfort in social situations, use of eye contact and facial expressions, and effective communication of ideas and emotions. However, these measures of social skills do not offer a conceptual framework to account for social skill development. In fact, a meta-analysis of social skills measures developed after 1994 concluded that the field would indeed benefit from a theoretical construct that accounted for social functioning and generated corresponding assessment instruments (Cordier et al., 2015).

Development of such a construct would require the incorporation of both domain-knowledge and the context(s) of the application. Although individual social skills may be rote and automatic, the contexts and rules for social interactions are dynamic (Silston et al., 2018). Unlike a number of motor skills that do not necessarily rely on domain knowledge (e.g., typing), social interactions require a wealth of domain knowledge, as well as knowledge about the specific context and individual(s) with whom one is interacting. This kind of social-skills framework would also need to account for how both the flexible application of skills and the problem-solving ability to make those decisions (i.e., intelligence) lead to social performance.

### Performance

Given that social performance is a primary metric of individual differences in social interaction, an understanding of how “performance” is conceptualized is worth considering. Performance has been defined as the extent to which a task is executed, or the recruitment of the behaviors and/or cognitive processes necessary to complete a task or goal (Posner, 1966). Performance has typically been defined operationally, based on the task being tested. In cognitive tasks, response times are often the primary index of performance [e.g., Stroop (Stroop, 1935) and Stop-Signal (Vince, 1948; Lappin and Eriksen, 1966; Logan and Cowan, 1984) tasks]. It is an open question, however, whether social performance is reflected more in maximal performance (or capability), or typical performance. Although particular social contexts may call for different levels of *effort* to attain a successful performance outcome (e.g., different degrees of effort expected of a job interview than meeting friends at a party), it is also the case that conscious awareness and attention to specific aspects of an interaction can actually impair outcomes (Alden and Wallace, 1995; Mueller et al., 2009; Asher et al., 2020). The discrepancy between effort and performance can also be seen when experts give conscious awareness to their performance (i.e., performance “choking”; e.g., Ericsson et al., 1993; Beilock and Carr, 2005; Logan and Crump, 2009; Macnamara et al., 2014; Logan, 2018). Maximum effort and maximum performance can therefore be expected to differ in the case of social interactions.

Applied to social interactions, maximum performance, distinct from effort, would then be the best possible social outcome an individual could attain, and typical performance would be the average social interaction outcome for that individual. This distinction could be reflected in the subjective depth and meaning of a single friendship (maximum performance) compared with subjective ratings of friendliness received from acquaintances (typical performance). However, measures of individual differences in social interactions rarely distinguish between maximum and typical performance. As a result, constructs conceptualized as abilities are assessed by measures of typical performance, pseudo-maximum performance *via* self-report inventories, or a mix of the two.

Typically, skilled performance has been measured in contexts in which there are clear performance outcomes. Better performance on a motor skill task is indicated by objective measures like speed of completion and/or task accuracy. However, in the social domain, speeded performance does not typically reflect successful performance, and accuracy is not as straightforward. It is difficult to say that a particular social interaction was correct or incorrect. A model of social performance that can accommodate adaptation to varying social contexts is therefore necessary.

### Expertise

Expertise is a conceptual framework, which is designed to integrate traits, abilities, and skills to account for domain-specific skilled performance. As discussed previously, intelligence-based constructs address the cognitive abilities necessary to learn skills. But these constructs do not account for the skills themselves that are used in an interaction, the distinction between maximum and typical performance, the need for hierarchical control, or social-interaction-specific experience. Those interested in individual differences in the social–emotional bases of interactions may benefit from research focused on individual differences in other domains of skilled performance. In particular, the application of domain-knowledge and experience to performance (i.e., “expertise”) has particular promise. Defined as the automatic, accurate, and holistic processing of domain-relevant stimuli (Diamond and Carey, 1986; Ericsson et al., 1993; Logan, 2018), expertise has been proposed to be the result of (deliberate) practice, with important influences from genetics and general cognitive ability (Ericsson and Lehmann, 1996; Ullen et al., 2016).

As discussed above, individual differences in social interactions have in the past been conceptualized as an intelligence, an essentially unwavering ability to problem-solve with social information. The assumption has then followed that social performance would be the result of this ability. Our contemporary understanding of brain function offers the opportunity for a more refined view of how social performance is achieved, and that addresses the highly dynamic nature of social interactions. Even the most socially gifted of individuals may be confronted with circumstances that lead to poor-quality interactions. As we continue to learn and model the functions of the brain, it has become increasingly clear that neural models that assume the brain is a predictive organ perform well for social tasks (e.g., Silston et al., 2018). In this approach, our brains constantly generate predictions about what will occur in our environments and update these predictive models based on prediction errors. Such prediction-error-based models have been proposed in emotion science (Hoemann et al., 2017; Gendron and Barrett, 2018; Hutchinson and Barrett, 2019), but have yet to be extended to social interactions. Social interactions demand a rapidly adaptable updating of predictions that can account for variation across context, the emotional state of the participants in the interaction, and the episodic priors that each individual is using to base their predictions of the current interaction. Further, these processes happen so quickly that hierarchical control is presumably necessary.

An intelligence framework can explain individual differences for a stable trait, and an intelligence-like parameter that sets the bounds for the cognitive flexibility utilized in social interactions is likely required. However, an intelligence-based theory is not sufficient to describe the complex decision-making and model updating that social interactions demand. An expertise framework addresses these needs. Expertise allows for the influence of prior experience (the episodic priors that influence the initial model and predictions about an interaction), the presence of hierarchical control, and an integration of context with the cognitive flexibility required to update predictions about interactions successfully. A social expertise would capture integration of the cognitive flexibility necessary to adapt quickly to the cues of a social partner based on prior experience, and then successfully translate that new model into social behaviors through a hierarchical control network. Individual differences in social interactions would thus vary based on (a) the level of cognitive flexibility required to shift among multiple neural nodes and integrate a host of sensory and internal information; and (b) prior experience with social interactions generally, and if the individual is an acquaintance, prior interactions with that specific individual. If one applies the instance theory of automaticity (Logan, 1988, 1995) and considers each social interaction as an instance in memory, then the number of social interactions engaged in, and the quality of encoded cues from those interactions, will over time dictate the automaticity with which the social information from an interaction is processed. The facility and accuracy of one’s models of social interaction will improve as a result, the variety of episodic priors (prior experiences that may resemble the current interaction) will be greater, and together will allow for a more accurate initial prediction and fewer prediction errors. If that processing can be generalized across instances in the social domain and done holistically, then suddenly social interaction processing starts to closely resemble other domains of expertise. Although an expertise framework has not previously been applied to individual differences in social performance, there is growing support for expertise as a means of conceptualizing individual differences in socio-emotional processes (e.g., emotion perception; Hoemann et al., 2020).

### Social–emotional expertise

Social–emotional expertise (SEE) is an individual difference construct that describes the ways in which individuals differ in the timing and quality of social interactions (McBrien et al., 2018; Wild and Bachorowski, 2020). Like the individual difference metrics described previously, SEE is intended to capture how individuals differ in social performance. SEE includes a theoretical orientation as to how social expertise develops, and testable hypotheses that derive from that orientation. An expertise framework of social performance may (a) explain how individual differences in social interactions develop, and (b) incorporate the constellation of traits and abilities that have already been identified as critical for social performance.

An expert utilizes a wide array of skills, traits, and abilities to accomplish a task within their domain of expertise. Specific attributes are utilized based on an individual expert’s baseline abilities and prior experience. We propose that expertise in social performance is similarly comprised of a combination of skills, traits, and abilities. Empathy, Openness, and EI are examples of such skills, traits, and abilities, respectively, that can be leveraged by the expert to attain high social performance (see Table 1).

**Table 1:** Definitions and examples of concepts relevant to individual differences in social performance.

Concept	Definition	Measures	Examples
Expertise	Automatic, accurate, and holistic processing of domain-relevant stimuli (Diamond and Carey, 1986; Ericsson et al., 1993; Logan, 2018)	Measures of holistic processing, accuracy, and automaticity	Vanderbilt expertise test
Performance	The extent to which a task is executed, or the recruitment of the behaviors and/or cognitive processes necessary to complete a task or goal (Posner, 1966)	Task-dependent	Response time on a cognitive task (e.g., stroop and stop-signal).
Ability	The maximal performance of an individual in a given situation (Carroll, 1993; Ackerman and Heggestad, 1997)	Performance-based measures	Intelligence test; academic achievement test
Trait	The typical performance of an individual in a given situation (Carroll, 1993; Ackerman and Heggestad, 1997)	Self and other report	Personality assessments
Intelligence	A global cognitive ability, encompassing the extent to which an individual can acquire, hold, and use information (Carroll, 1993; Neisser et al., 1996; Jensen, 1998; Schneider and McGrew, 2018)	Ability-based measures	Wechsler adult intelligence scales (WAIS)
Skills	Behaviors that are demand-specific and have a degree of correct and incorrect outcomes (McDonald, 1965; Welford, 1980; Downing, 1982)	Ability-based and trait-based measures	Riggio social skills inventory
Social intelligence	The ability to “understand and behave wisely” in interactions with others; social problem solving (Sternberg and Smith, 1985; Cantor and Kihlstrom, 1986; Kihlstrom and Cantor, 2000)	Trait-based measures	Tromso social intelligence scale
Emotional intelligence	The ability to “accurately understand the emotions of one’s self and others,” and to use emotional information to influence one’s own thinking and thoughts of others (Salovey and Mayer, 1990)	Ability-based [e.g., Mayer-Salovey-caruso emotional intelligence test (MSCeiT)] and trait-based (e.g., schutte emotional intelligence scale)	MSCEIT (four factors: emotion perception, facilitating cognition through emotion, understanding emotion, and managing emotion)
Social–emotional expertise	The ways in which individuals differ in the timing and quality of social interactions (McBrien et al., 2018; Wild and Bachorowski, 2020)	Multi-method	SEE scale

The self-perceived social performance component of SEE is currently measured with the SEE Scale (McBrien et al., 2018); a 25-item self-report measure that assesses perceived typical performance in social interactions. SEE Scale scores have been associated with higher social interaction quality ratings by naïve outside observers (Wild and Bachorowski, 2020), and greater positive affect induced in third party observers by individuals with high SEE Scale scores (Wild and Bachorowski, 2020). These results indicate that the SEE Scale is able to capture aspects of an individual’s characteristics that have an impact on the quality of the social interactions they undertake. As mentioned previously, experience is a crucial factor in social interactions. A feature of individuals’ self-report of their typical social interaction performance *in general* is that SEE Scale scores will likely shift over time and experience in specific social contexts. Given more experience in a specific social context, one would expect to gain skill and perceived competence (or the belief that one is capable of effectively operating in a given context), improving SEE Scale scores. The SEE Scale is meant to be a self-reported index of overall social performance, which is reflected in the general wording of items (e.g., “I can easily draw on my various social skills as situations warrant”) and the directions (“Please answer each of the following items by circling the response that best describes what’s typical of you.”). However, it is also possible that SEE may be better modeled as an expertise specific to contexts, such as expertise in professional contexts differing from expertise in casual contexts, with limited transfer from one context to another. In other words, it has yet to be determined whether social performance from one context to another is better modeled as near or far transfer (e.g., Kassai et al., 2019).

In this way, the social expertise approach allows for a theory driven means of explaining improvement in social skills that lead to improved social performance, a well-supported mechanism of intervention for social interaction improvement, while incorporating individual differences constructs (e.g., emotion perception and other components of intelligence frameworks). The SEE framework also offers an alternative approach to addressing the criterion problem by asking individuals to rate their typical qualitative performance related to various social-interaction-relevant scenarios (e.g., “I can easily draw on my various social skills as situations warrant”), perceptions (e.g., “I’m good at reading facial expressions”), and behaviors (e.g., “I’m good at making eye contact.”). When paired with more objective measures of these perceptions and behaviors, a construct validation (*via* a nomological network) that can accommodate the complexities of social interactions becomes more feasible. The goal thus becomes to develop optimal, objective measures of socially relevant perception and behavior.

In pursuit of a more thorough and broad-ranging assessment of social performance as an expertise (i.e., the SEE framework), varied methods of measurement must be considered beyond the self-reported SEE Scale. Measurement of individual differences in social interactions, like individual differences more broadly (e.g., object recognition, see Richler et al., 2019), has typically relied on single measures of each construct of interest at a single timepoint. As Richler et al. (2019) described, such an approach is problematic when behavioral outcomes are of interest, as correlations among time points and across contexts are typically low and thus minimize the stability and magnitude of the correlations that can be detected. Additionally, correlations may be further constrained by different levels of experience among participants with a given socio-emotional performance context, as has been an issue in nonsocial research. Further work that assesses individual differences in social interactions with multiple measures of each construct included, in the same sample, can begin to unify the existing constellation of constructs and combine them within an expertise framework.

## Quantifying expertise in social–emotional contexts

In order to address the measurement needs of a SEE framework, we next consider the primary components of expertise (i.e., working memory, automaticity, and holistic processing) in social contexts and how best to assess them. Automaticity, or the performance of a behavior automatically (i.e., without top-down control), has been demonstrated with social stimuli (e.g., emotion recognition; Elfenbein and Ambady, 2003). Similarly, holistic processing, or attentional focus on the domain-relevant features of a stimulus, has also been demonstrated through face perception tasks and its disruption in social contexts (e.g., social anxiety disorder; Mueller et al., 2009; Curby et al., 2012). As a result, there is evidence to support the perception of social stimuli as an automatic and holistic process. Automaticity and holistic processing with respect to SEE have been discussed elsewhere (Wild and Bachorowski, 2020) as have related social–emotional trait and abilities that may contribute to SEE (McBrien et al., 2018; Wild and Bachorowski, 2020). Therefore, the foci of the current discussion are working memory in SEE and potential means for quantifying SEE.

### Social–emotional working memory

For individual differences in social interactions to function as an expertise, there must be a means for automatic and effortful cognitive processes to be integrated. In order to achieve this integration, information must be held and manipulated in working memory. Working memory has been identified as an important component of skilled performance, with experts (e.g., chess experts) demonstrating increased working memory capacity for items in their domain of expertise (e.g., positions on a chessboard; Ericsson and Kintsch, 1995). The increase in working memory capacity associated with skilled performance is proposed to be the result of the utilization of long-term memory in conjunction with retrieval cues held in working memory (Ericsson and Kintsch, 1995). Higher overall working memory capacity is also associated with expert performance, above and beyond differences in experience (Meinz and Hambrick, 2010), and domain-specific working memory enhancement is not temporary or dependent on active rehearsal (Ericsson and Delaney, 2004).

Given working memory’s central role in expertise in other domains, we expect working memory to also be important in social expertise. Predictions generated in prefrontal cortical regions and sensory information from other brain areas must be combined in order for information from extero- and interoceptive cues to update predictions about the environment (Silston et al., 2018; Lockwood et al., 2020). Other studies have shown an increase in visual short-term memory for experts in their domain of perceptual expertise (Moore et al., 2006; Curby et al., 2009). It is clear that experts have a greater capacity to hold information about domain-specific stimuli in working memory, and thus have greater capacity to integrate a wide array of information regarding domain-specific stimuli. It is expected, then, that social experts will show greater working memory capacity for social stimuli. In support of this hypothesis, recent work in EI has identified an emotion-information processing factor that may be analogous to a social working memory (Fiori et al., 2022).

There is also evidence to suggest that social expertise may use a neural network similar to that described for expertise in other domains (Meyer et al., 2012, 2015). For example, work has shown that activation in an expertise-related network has been associated with response to social working memory load specifically, and predicts performance on a difficult perspective-taking task (Meyer et al., 2015). Other work investigating the unique contribution of social information has found that social information is held more efficiently in working memory than nonsocial information (Thornton and Conway, 2013).

These studies have found significant effects with a single trait measure of either perspective-taking or trustworthiness. A further test of expertise as an effective theoretical framework would be to develop a robust battery of measures of social expertise and assess the extent to which performance on this battery correlates with social-working-memory-related neural activation. Additionally, work that incorporates the social working memory capacity of an individual into predictions of their social functioning would provide clarity on the specific role that social working memory plays in social performance. Outcomes would have particular relevance to whether the level of experience (i.e., practice) that an individual has with social situations predicts social performance when controlling for a more general social working memory capacity. This differentiation has been seen in other areas of expertise, such as piano sight-reading (Meinz and Hambrick, 2010).

### Quantifying SEE

As detailed in this review, individual differences in social interactions have been quantified in numerous ways, including through observer ratings, self-report, and tests of accuracy. Self-perceived SEE is currently measured with a reliable and valid self-report scale (McBrien et al., 2018). Similar to self-report measures of expertise in other domains (e.g., vision; Richler et al., 2014), the SEE Scale is able to capture one aspect of this overall expertise. However, multimethod approaches, utilizing assessments of traits, abilities, and skills relevant to social performance, could be used to more fully define social expertise. Given the significant correlations among many social performance-related measures, it would be informative to test the latent structure of these measures. Factor analysis and structural equation models with a large set of social performance measures can be used to identify the latent factors being tapped by measures of individual differences in social interactions (including the SEE Scale). Additionally, a structural equation modeling approach could elucidate the relations among various latent factors of social performance. This approach would also allow for a determination of an underlying general factor of social performance, as has been undertaken for visual ability (Gauthier et al., 2014; Richler et al., 2019) and psychopathology (Lahey et al., 2012).

In concert with identifying the latent constructs underlying social performance measures, multimethod assessments of holistic processing, automaticity, and accuracy of participants’ expertise in the social–emotional domain are needed. One potential avenue to address this need is the development of a social performance-based task within an expertise framework, analogous to the Cambridge Face Memory Test (CFMT; Duchaine and Nakayama, 2006) and the Vanderbilt Expertise Test (VET; McGugin et al., 2012). These tests use training on exemplars from the specific domain(s) of interest (e.g., faces, cars), and then test memory for those exemplars with a series of items from each category that are not identical to the exemplars but share the same category. The VET in particular, as it contains eight distinct categories of visual objects, allows for comparisons between domain-specific and domain-general knowledge. A conceptually similar expertise task in the social domain could include social interactions (the domain of expertise), as well as interactions with familiar and unfamiliar inanimate objects such as avatars (tangential to the domain of expertise). Parametric assessment of a variety of interactions would reveal the extent to which individuals become expert in interactions with other people, and where along the gradient of anthropomorphic attributes experts are able to process interactions holistically, automatically, and accurately. Novel objects also allow some control for experience, which can affect the magnitude of the correlations among measures (as discussed previously).

The issue of accuracy in social interactions (i.e., the “criterion problem”) is an important one. Unlike correctly identifying a car, there are typically not binary, correct-incorrect responses to social interactions. Instead, social interactions arguably take place along a continuum of quality. Rather than relying on correct-incorrect responses to social questions, social expertise measures should instead emphasize the means by which individuals *process* social information. Much like chess experts, who can win a chess game through multiple, different combinations of moves, social experts might also achieve high quality interactions *via* disparate combinations of behaviors that nonetheless share common processes. Attempts to quantify social expertise will thus be bolstered by (a) accounting for processes (e.g., hierarchical control), in addition to behavior, and (b) testing whether processing of social information reflects the holistic, automatic, and accurate approach of an expert.

## Future directions for SEE

In this final section, we focus on the specific methods by which the measurement and modeling of SEE could be developed. The goal is to demonstrate how the principles described thus far can be used to inform not only further work with SEE, but also the ongoing development of social performance constructs more broadly. Specific attention is given to (a) the importance of measuring dyadic interactions, and (b) computational modeling approaches that can account for the complexity of social interactions.

### Importance of the interactional unit

The assessment of social interaction performance must be grounded in assessment of the unit of interaction (e.g., the dyad or triad of individuals interacting), as social interactions are not individual-level phenomena, but instead take place at the unit of a group (e.g., dyads; see Kenny and Albright, 1987; Bernieri and Gillis, 2001; Kenny et al., 2006). Recent work has shown the importance of assessing and testing the social interaction performance of dyads rather than individuals (Goldring and Bolger, 2022). Ample evidence also exists to demonstrate that emotions, including mood and affect, are experiences with inherently interactive aspects (e.g., Uchida et al., 2022). Future work focused on the assessment of social performance as an expertise will benefit from examining the ways in which social performance (a) varies across social contexts in tandem with (b) specific interactions and interactional partners. In such examinations, the group-level outcomes of social performance, such as shared affect, stress, or mood, can be tracked for each individual across that individual’s interactions. Patterns of performance can then be determined, offering a detailed assessment of an individual’s social expertise.

### Modeling social interactions

Social interaction-related skills, abilities, and expertise are assessed through a broad array of dimensions that are themselves represented by a host of constructs. One barrier to effectively integrating social performance assessments is the difficulty in characterizing individuals across multiple performance-related dimensions simultaneously. Although regression or dimension-reduction techniques (e.g., structural equation modeling) offer robust methods for the distillation of social performance measures into generalizable metrics, these methods are less robust for capturing the dynamic nature of the interrelations among multiple dimensions (e.g., skill, ability, and expertise) of an individual’s actual social performance. To do so requires alternative strategies for modeling individual-level social performance.

**Figure 2:**
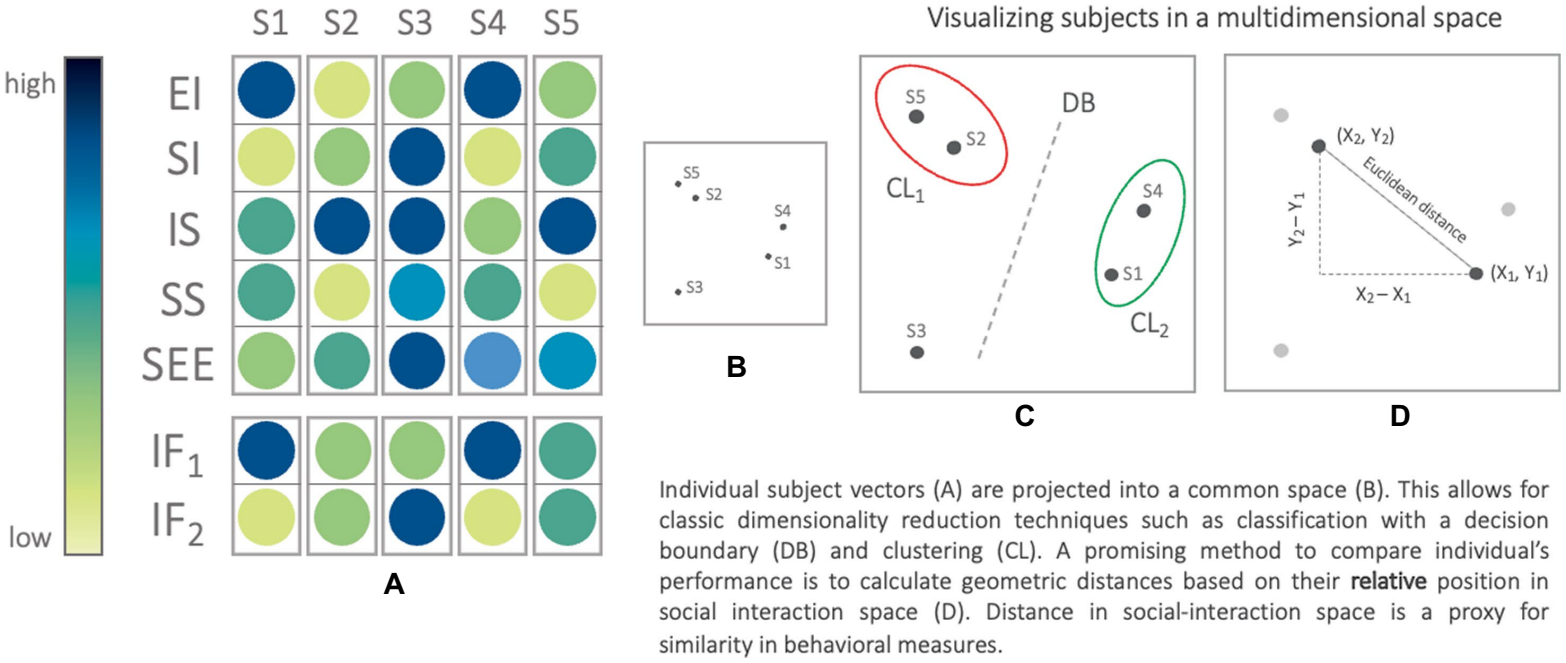
Schematic demonstrating the process of using individual vectors derived from measures of social performance **(A)** to create a social performance space **(B)** in which individuals can be clustered **(C)** or compared **(D)** using Euclidean distance.

One promising avenue in this regard is the use of multidimensional computational modeling (See Figure 2). This general approach has similarly been proposed for improving precision in other complex domains, such as semantic memory (Cutler et al., 2019) and psychiatry (Wiecki et al., 2015). To create such a model, a host of features (Figure 2A) are set as dimensions in a multidimensional space, and individuals are then located as unique positions within this space (based on their placement along these dimensions). To apply this to the example of social interaction performance, an individual has a host of measurable features (e.g., EI, SI, interpersonal sensitivity, social skills, and SEE; see Figure 2A). Interpersonal factors, such as warmth (IF_1_ in Figure 2A) and competence (IF_2_ in Figure 2A), could also be included as features in such a model (Seewald and Rief, 2022). Individuals are assigned a feature vector based on their responses to the assessed social performance metrics, creating a set of coordinates that point to a unique “address” in the *n*-dimensional space. The distance between “addresses” represents the (dis)similarity between individuals’ responses. The strength of that relationship can be quantified using similarity measures like Pearson’s correlation, or distance metrics like Euclidean distance or cosine similarity (Ontañón, 2020; see Figure 2D). Identical responses share an address, similar responses are nearby, and distinct responses place an individual farther in social performance space.

The end result of this process could be precision modeling of individual-level social performance attributes. Outcomes can then be compared across individuals to identify patterns of performance, predict interactional outcomes, and understand the development of social skills over time. In this way, the complexity of an individual’s social performance and related attributes can be quantified using existing assessments, and represented in a common social-performance space that captures the dynamic nature of individual differences (Schafer and Schiller, 2018; Hayman and Arzy, 2021).

Further, individuals have unique combinations of identities and characteristics that go beyond specific social skills and abilities but can nonetheless impact social performance. It is well established that aspects of individual identity (e.g., age, race, ethnicity, urban/rural, and SES) impact social interactions (e.g., Filardo, 1996; Wang et al., 2020; McKone et al., 2021; Wang et al., 2021). However, to our knowledge, no currently utilized approach to modeling social performance is able to account for these factors. Using high-dimensional modeling, an unlimited number of features can be added or removed, allowing researchers to include critical, identity-related data—like age, race, and SES—that might contribute to an individual’s social performance. Such an approach also allows for integration of data across multimethod assessments of socially relevant factors (e.g., social working memory, automaticity, self-reported traits, and observer reports). The flexibility to merge multiple measures into a single representative response vector allows researchers to select metrics that explain the greatest variance, and simulate or predict performance by changing values for features of interest.

Our multi-dimensional, social-interaction model is widely applicable because it is both descriptive and predictive. We can capture an individual’s social skills in the explanatory model, and use that descriptive data to simulate alternative outcomes in the predictive model. Combining the model’s descriptive and predictive ability allows for two types of important comparisons: we can compare across individuals (between-subjects), and we can compare within individuals across contexts.

A between-subjects analysis calculates the distance between two vectors in social-performance space to quantify the similarity of individuals in how they navigate social interactions. We can then treat each feature as a variable, and by simulating new numbers for that variable, we can predict where that individual would be in social-interaction space if one feature was changed. For example, the social interaction performance could be predicted for a physician based on varying levels of features (e.g., SEE, EI, empathy, or gender identity) in a prospective patient to find what features the physician will need to adapt to create the best patient-provider fit. There is also the possibility of comparing an individual to a group. A benefit of vector space models is in their flexibility to represent one instance, or the average of instances (e.g., one person or a “typical person”; Logan, 1988, 1995). Group vectors can be additive (the summation of individual vectors), or average (the mean of individuals), and remain in the same space because each element still represents one measure. It is therefore possible to create an “ideal” or “goal” social-interaction skill-set, and vary the elements of one individual to gain insight as to what behavioral changes are needed to work toward that goal.

A within-subjects analysis elevates the model from one that describes or explains social performance, to one that can predict the interactions of an individual in a new context. As an example, the same person will interact differently in a physician’s office than meeting friends at a party. By adding “context” elements to a vector, we can place people in a social space that truly reflects the dynamics of social engagement, and predict how they would adaptively interact in a new, unmeasured context based on prior locations in social space.

Creating a high-dimensional space that represents social performance is therefore beneficial because it transcends any one theoretical framework. Individuals are represented as a unique combination of cognitive, clinical, and identity measures and evaluated based on their relative similarity to others in this purely data-driven approach to social dynamics. In doing so, an individual’s experience is more accurately captured by reducing the biases of researchers regarding what aspects of identity are most relevant to social interactions.

## Summary

Social interaction performance is an individual difference that a broad range of constructs and measures have attempted to capture, ranging from intelligence-based theories to skills-based inventories agnostic to theory. Intelligence frameworks have been particularly prominent for conceptualizing the individual differences of interest, but the means by which social or emotional intelligence is assessed have varied considerably in reliability and construct validity. Measurement issues in current assessments of individual differences in social interactions are well-documented, with the criterion problem in social performance, the presence of inconsistencies between definition and measurement, and the need for mixed/multiple method designs for improving construct validity of social–emotional constructs of particular note.

We propose that an expertise framework offers a mechanism by which experience and ability can be combined into an explanation of social performance. An expertise approach may mitigate measurement issues by utilizing measures on which skilled performance is the primary outcome. Efficient, accurate processing of social information and the prediction of social interaction quality from that processing addresses the criterion problem in a novel way, by being grounded in knowledge about expert performance accrued from knowledge in other domains. The similarities in what is already known about individual differences in social performance and what is known about performance in other domains of expertise provide preliminary indications that an expertise framework may prove effective as a unifying framework. The exciting potential for the use of prediction/prediction-error based approaches for modeling social performance, as is being done in related areas, including emotion science, offers additional motivation for pursuing expertise as a framework for social performance. Finally, approaches that stem from computational neuroscience and computational psychiatry to integrate the wide array of assessments of social performance-related constructs offers a precise means for modeling social interaction performance. The resulting outcomes hold promise for translational applications of psychological theory that could benefit the quality of interventions in both psychological and medical settings.

## Author contributions

MW: conceptualization, methodology, software, formal analysis, investigation, writing–original draft, writing–review and editing, visualization, and funding acquisition. RC: conceptualization, writing–review and editing, and visualization. J-AB: conceptualization, writing–review and editing, supervision, and funding acquisition. All authors contributed to the article and approved the submitted version.

## Funding

This work was supported in part by a Graduate Research Fellowship from the National Science Foundation. This material is also the result of work supported with resources and the use of facilities by the Veterans Affairs Office of Academic Affiliations Advanced Fellowship Program in Mental Illness Research and Treatment, the Central Texas Veterans Health Care System, and the VISN 17 Center of Excellence for Research on Returning War Veterans, Waco, TX, United States.

## Conflict of interest

The authors declare that the research was conducted in the absence of any commercial or financial relationships that could be construed as a potential conflict of interest.

## Publisher’s note

All claims expressed in this article are solely those of the authors and do not necessarily represent those of their affiliated organizations, or those of the publisher, the editors and the reviewers. Any product that may be evaluated in this article, or claim that may be made by its manufacturer, is not guaranteed or endorsed by the publisher.

## Authors disclaimer

The views expressed in this manuscript are those of the authors and do not necessarily reflect the position or policy of the Department of Veterans Affairs, or the United States Government.
